# NOX4 Signaling Mediates Cancer Development and Therapeutic Resistance through HER3 in Ovarian Cancer Cells

**DOI:** 10.3390/cells10071647

**Published:** 2021-06-30

**Authors:** Wen-Jing Liu, Ying-Xue Huang, Wei Wang, Ye Zhang, Bing-Jie Liu, Jian-Ge Qiu, Bing-Hua Jiang, Ling-Zhi Liu

**Affiliations:** 1 School of Basic Medical Science, Academy of Medical Science, The Affiliated Cancer Hospital of Zhengzhou University, Zhengzhou 450001, China; 15238311663@163.com (W.-J.L.); yingxue1128@163.com (Y.-X.H.); 18339200129@163.com (W.W.); zlyyzhangye2393@zzu.edu.cn (Y.Z.); bingjieliu@zzu.edu.cn (B.-J.L.); 2 Department of Pathology, Anatomy and Cell Biology, Thomas Jefferson University, Philadelphia, PA 19107, USA; 3 Department of Medical Oncology, Thomas Jefferson University, Philadelphia, PA 19107, USA; Ling-Zhi.Liu@jefferson.edu

**Keywords:** NOX4, HIF-1α, HER3, therapeutic resistance, ovarian cancer

## Abstract

Development of resistance to therapy in ovarian cancer is a major hinderance for therapeutic efficacy; however, new mechanisms of the resistance remain to be elucidated. NADPH oxidase 4 (NOX4) is responsible for higher NADPH activity to increase reactive oxygen species (ROS) production. In this study, we showed that higher levels of NOX4 were detected in a large portion of human ovarian cancer samples. To understand the molecular mechanism of the NOX4 upregulation, we showed that NOX4 expression was induced by HIF-1α and growth factor such as IGF-1. Furthermore, our results indicated that NOX4 played a pivotal role in chemotherapy and radiotherapy resistance in ovarian cancer cells. We also demonstrated that NOX4 knockdown increased sensitivity of targeted therapy and radiotherapy through decreased expression of HER3 (ERBB3) and NF-κB p65. Taken together, we identified a new HIF-1α/NOX4 signal pathway which induced drug and radiation resistance in ovarian cancer. The finding may provide a new option to overcome the therapeutic resistance of ovarian cancer in the future.

## 1. Introduction

Ovarian cancer is one of the leading causes of cancer-associated death among women. About 70 percent of patients with ovarian cancer were found to be in advanced stages (Stages III, IV), chemotherapeutic resistance and tumor recurrence will always occur after a period of treatment [1]. In the past 10 years, the prevalence rate of ovarian cancer has significantly increased, but the therapeutic effect has not significantly improved [2]. Therefore, there is an urgent need to find new and tolerable effective methods for the treatment of ovarian cancer.

Increased reactive oxygen species (ROS) production has been detected in various cancers and thought to be oncogenic, they were reported to cause genomic instability and DNA damage [3], contributing to the activation of pro-survival signaling pathway, loss of tumor suppressor gene-function, and increasing glucose metabolism, resulting in cancer progression and drug resistance [4]. In our previous study, we found that ROS levels were significantly increased in ovarian cancer cells compared with normal cells, which regulated hypoxia-inducible factor 1α (HIF-1α) and vascular endothelial growth factor (VEGF) expression, and induced angiogenesis and tumor growth [5]. NADPH oxidase 4 (NOX4) was responsible for higher NADPH activity to increase ROS production, and NOX4 mRNA levels were much higher in ovarian cancer cells than those in normal cells [5]. However, the molecular mechanisms leading to higher levels of NOX4 in ovarian cancer cells remain to be elucidated.

HER2 (ERBB2) monoclonal antibody trastuzumab has achieved exciting results in the treatment of breast cancer. Considering that of 9–30% of ovarian cancer patients were HER2 positive, and there are several similarities of the biology of HER2 between breast cancer and ovarian cancer, it would be feasible to treat ovarian cancer via HER2 target therapy [6,7]. However, one Phase II clinical trial confirmed that trastuzumab was not effective enough in the treatment of ovarian cancer [8], and the reasons were not clear yet. Our previous study showed that high levels of ROS significantly increased the expression levels of HER2 and HER3 in ovarian cancer [3]. Therefore, it is interesting to further explore whether NOX4 is involved in the therapy resistance of ovarian cancer cells.

In this study, we first verified that NOX4 expression levels were elevated in ovarian cancer tumor tissues. To understand the molecular mechanism of NOX4 upregulation, we showed the role of HIF-1α in mediating the alternative splicing of NOX4 expression. Furthermore, we demonstrated that NOX4 played a pivotal role in targeted therapy and radiotherapy resistance in ovarian cancer cells. Finally, we identified HER3 and NF-κB p65 were down-stream molecules in NOX4-mediated drug resistance. This work would provide an experimental basis for further elucidating the molecular mechanism of ovarian cancer treatment and prognosis judgment in the future.

## 2. Materials and Methods

### 2.1. Cell Culture and Transfection

Human ovarian cancer cell lines A2780 and OVCAR3 (kindly provided by Dr. Jing-Jie Yu at West Virginia University, Morgantown, WV, USA) were cultured in DMEM medium supplemented with the following components: penicillin (100 units/mL), streptomycin (100 units/mL) and 10% FBS. Cells were placed at 37 °C incubator with 5% CO_2_. The cell transfection was performed using X-treme GENE HP DNA Transfection Reagent (Roche, Mannheim, Germany) by following the manufacturer’s instruction. These cells were cultured in the presence of 1 μg/mL puromycin for 3–4 weeks to select stable cell lines.

### 2.2. Clinical Ovarian Cancer Specimens

The human ovarian tumor tissues and adjacent normal tissues of ovarian cancer patients were obtained from the tissue bank of the Affiliated Cancer Hospital of Zhengzhou University (Zhengzhou, China). The ovarian cancer tissue had been collected and stored in the tissue bank for several years. The patient information including names, age and other patient personal information was not known to the investigators. Based on the coded information, the patients had high-grade serous cell carcinoma (HSCC), but no history of other tumors and no autoimmune diseases. The patients did not receive surgical treatment, radiotherapy, chemotherapy or immunization inhibitory therapy before surgery.

### 2.3. Compounds and Plasmids

GKT137831 (S7171) and afatinib (S1011) were purchased from Selleck (Selleck, Houston, TX, USA), and dissolved in DMSO for stock, and diluted with PBS. All shRNA plasmids were purchased from Genechem. (Genechem, Shanghai, China). HER2/HER3 cDNA plasmids and HIF-1α knockout plasmids were designed by our laboratory.

### 2.4. RNA Extraction and Quantitative Real-Time PCR

Total RNAs were extracted and purified using TRIzol reagent (10296010, Thermo Fisher Scientific, Waltham, MA, USA) by following manufacturer’s instruction. The first-strand cDNAs were synthesized by 1 μg total RNAs (R323-01, Vazyme, Nanjing, China) and mixed with specific primers, RNase-free water, and ChamQ Universal SYBR qPCR Master Mix and detected the mRNA expression levels of NOX4 and NOX4C via a real-time PCR system (Q711-03, Vazyme, Nanjing, China). The relative levels of specific RNAs were measured and normalized to expression levels of GAPDH (an internal control). Primers used were as follows:

NOX4-F Ex1: CCATGGCTGTGTCCTGGAGGAGCTG;

NOX4-R Ex18: CACAGCTGATTGATTCCGCTGAG;

NOX4C-F Ex1: CCATGGCTGTGTCCTGGAGGAGCTG;

NOX4C-R Ex12/8: CAGTTGGACACCTGAAACATG;

GAPDH-F: TGAACGGGAAGCTCACTGG;

GAPDH-R: TCCACCACCCTGTTGCTGTA.

### 2.5. Western Blotting

Cells were lysed in cell lysis buffer and cell lysate supernatants were obtained by centrifugation at 13,000× *g* at 4 °C for 15 min. Aliquots of total proteins (30–40 µg) were used to perform immunoblotting analysis using the following antibodies: rabbit anti-NOX4 (1:1000, ab133303, Abcam, Waltham, MA, USA), rabbit anti-HIF-1α (1:1000, BS3514, Bioword, Nanjing, China), rabbit anti-HER2 (1:1000, 18299-1-AP, Proteintech, Wuhan, China), rabbit anti-HER3 (1:1000, 10369-1-AP, Proteintech, Wuhan, China), rabbit anti-NF-kB p65 (1:1000, ab16502, Abcam, MA, USA), rabbit anti-β-actin (1:5000, AP0060, Bioword, Nanjing, China) and rabbit anti-GAPDH (1:5000, AP0063, Bioword, Nanjing, China).

### 2.6. Cell Viability Assays

Cells were seeded in 96-well plates and cultured overnight. The cell viability was determined 72 h after drug treatment using the CCK-8 Kit (Dojindo Laboratories, Kumamoto, Japan) according to the manufacturer’s instruction. Briefly, 10 μL of CCK-8 solution were added to each well, followed by incubation at 37 °C for 1 h. The OD values were measured at 450 nm using a microplate reader.

### 2.7. Radiation and Clone Formation

Stable cell lines were irradiated with 6MV X-ray linear accelerator at doses of 0 Gy, 2 Gy, 4 Gy and 6 Gy. The dose rate was 200 Gy/min, the source-target distance was 100 mm. After irradiation, the cells were digested with 0.25% trypsin and counted. Cells were plated in 6-well plate at corresponding irradiation doses, and plated in 37 °C, 5% CO_2_ incubator for 10–14 days. The numbers of cells were 300 (0 Gy), 600 (2 Gy), l000 (4 Gy) and 5000 (6 Gy)/hole as indicated. Then cells were stained with 1% crystal violet solution, the numbers of cell clones formed in each hole were counted (clones > 50 cells, count as 1 clone). Clone formation rates (PE) and cell survival rates (SF) were calculated, PE = (number of clones in blank control group/number of cells plated in blank control group) × 100%, SF = number of clones in irradiated experimental group/(number of cells plated in blank control group * PE) at a certain dose.

### 2.8. Luciferase Reporter Assay

Cells were seeded in 24-well plates overnight and transfected with 1 μg promoter luciferase reporter vector and 0.1 μg renilla luciferase expression vector (pGL4.74). Cells were harvested 48 h after transfection, and luciferase activities were detected and analyzed according to the manufacturer’s instructions (E1910, Promega, Madison, WI, USA).

### 2.9. Database

NOX4 expression levels were detected and analyzed using the cancer genome atlas (TCGA) dataset. The prognostic values of NOX4 mRNA expression levels were evaluated using an online database, Kaplan–Meier Plotter. The overall survival (OS) and progression free survival (PFS) were obtained from the TCGA dataset of NOX4 via probe 236843.

### 2.10. Statistical Analysis

All calculations were performed using GraphPad Prism 8.0 and presented as mean ± SEM. If there are only two groups, we analyzed the data by a paired Student’s *t*-test for paired data or Student’s *t*-test for unpaired data. The difference was considered significant at *p* < 0.05. Survival curves of our data were plotted using Kaplan–Meier curve and compared using the log-rank test.

## 3. Results

### 3.1. Higher NOX4 Levels Were Correlated with Ovarian Cancer Development and Poor Progression-Free Survival

**Figure 1:**
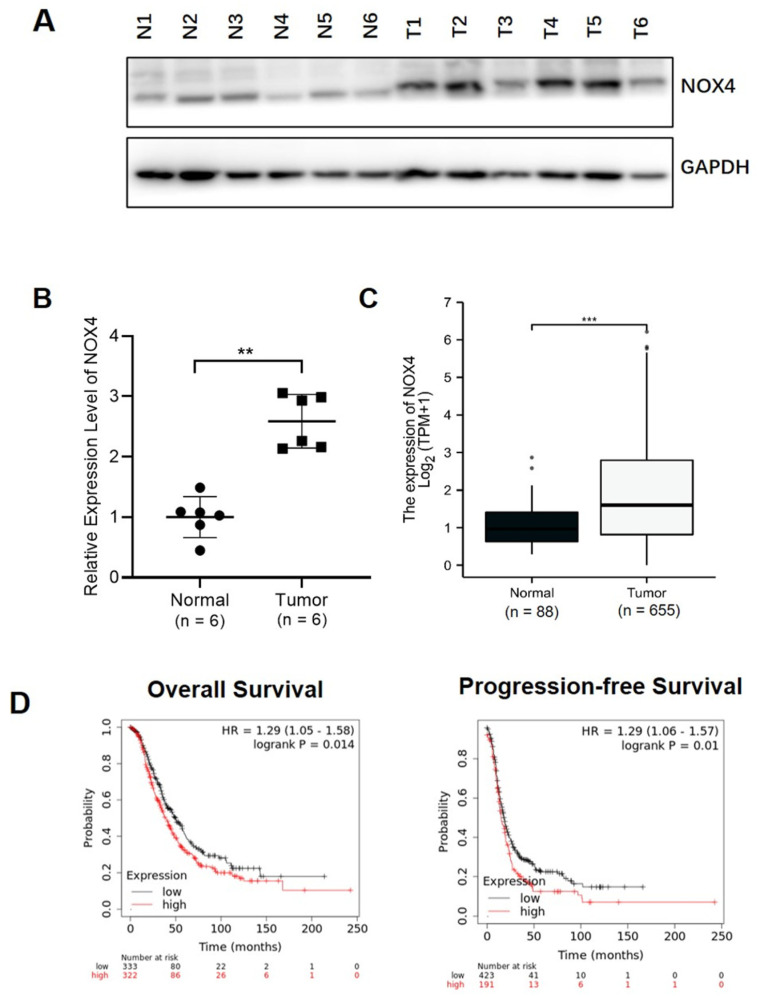
High NOX4 levels were correlated with ovarian cancer development. (**A**) The expression levels of NOX4 in tumor tissues of human ovarian cancer patients (N1–N6) and the normal control samples (T1–T6). (**B**) The mRNA expression levels of NOX4 in ovarian cancer tissues and normal tissues obtained from the TCGA database. (**C**–**D**) The correlation between NOX4 expression levels and overall survival (OS) or progression-free survival (PFS) in ovarian cancer patients in the TCGA database. ** Indicates significant difference at *p* < 0.01, *** Indicates significant difference at *p* < 0.001.

In this study, we detected the expression levels of NOX4 in six pairs of human ovarian cancer tissues and adjacent normal tissues. Our results showed that the expression levels of NOX4 were significantly higher in the tumor tissues compared with adjacent tissues. The representative results from the tissues were shown in Figure 1A. Consistently, analysis of NOX4 mRNA expression levels in the TCGA database showed that NOX4 expression levels were significantly higher in ovarian cancer tissues than those in normal tissues (Figure 1B). The Kaplan–Meier curve and log-rank test analyses revealed that NOX4 mRNA levels were strongly associated with the overall survival (OS) and progression-free survival (PFS), and higher levels of NOX4 mRNA were significantly associated with the lower OS and PFS in the ovarian cancer patients in the TCGA database (Figure 1C).

### 3.2. Expression Levels of NOX4 Were Regulated by HIF-1α via Alternative Splicing in A2780 Cells

**Figure 2:**
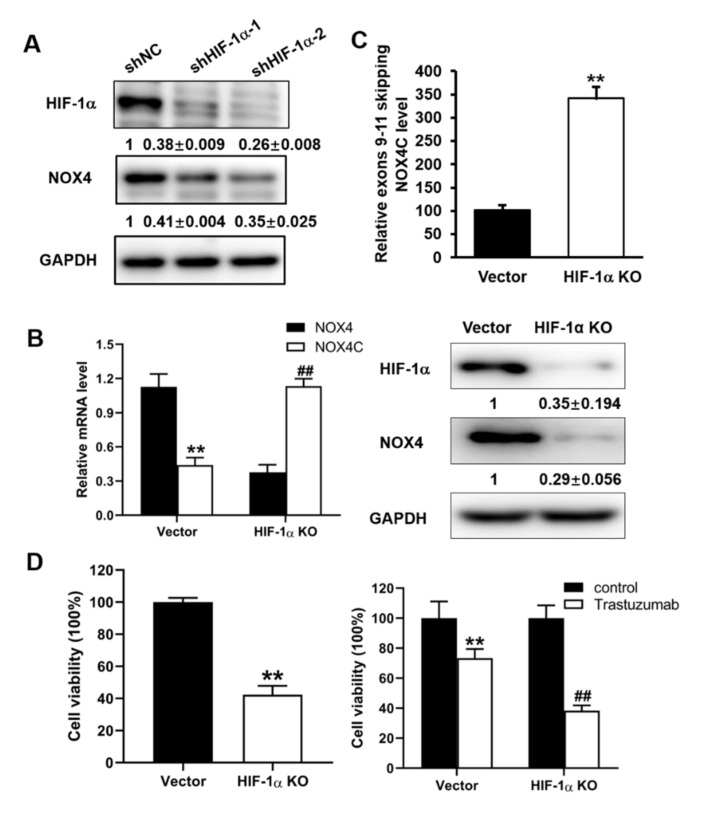
Expression levels of NOX4 were regulated by HIF-1α via alternative splicing in A2780 cells. (**A**) Knockdown the expression level of HIF-1α in A2780 cells via the shRNA of HIF-1α (shHIF-1α-1 and shHIF-1α-2) and the negative control (shNC), the expression level of NOX4 and HIF-1α were detected and the respective pictures and the quantitative data (SEM ± SD, n = 3) are shown. (**B**) NOX4 mRNA and protein expression level in HIF-1α knockout A2780 cells detected by RT-PCR and Western blotting. (**C**) The exons 9–11 skipping NOX4C expression levels in HIF-1α knockout cells. (**D**) Cell viability and response to trastuzumab in HIF-1α knockout A2780 cells. ** and ## indicates significant difference at *p* < 0.01.

NOX4 has different isoforms produced by alternative splicing for regulating gene expression [9]. In addition to the full length functional NOX4, the non-functional, dominant negative isoform NOX4C was produced by cutting off exons from 9 to 11 containing NAD(P)H/FADH binding regions, resulting in the loss of function [10]. Because ovarian cancer tissues were known to be hypoxia with higher levels of HIF-1α expression [1,2], and, as shown in Supplementary Figure S1, the HIF-1α expression levels were high and were strongly correlated with the overall survival of ovarian cancer patients. Therefore, we first investigated whether NOX4 was regulated by HIF-1α in A2780 ovarian cancer cells. Cells were transfected with shHIF-1α and control vector shNC, Western blot analysis revealed that knockdown of HIF-1α significantly decreased the full length and functional NOX4 expression (Figure 2A). Furthermore, knockout HIF-1α by CRISPR-cas9 resulted in significant downregulation of NOX4 in A2780 ovarian cancer cells (Figure 2B). These results suggested that HIF-1α regulated the expression levels of NOX4.

As HIF-1α regulated the alternative splicing of adrenomedullin pre-mRNA [11], we investigated whether HIF-1α may regulate the splicing of NOX4. Our results indicated that HIF-1α knockout greatly increased NOX4C mRNA expression levels in A2780 (Figure 2C). We then detected the cell viability and response to trastuzumab in HIF-1α knockout A2780 cells, and our results showed that HIF-1α knockout significantly decreased the cell viability and increased the therapy sensibility to trastuzumab (Figure 2D). These results suggested that HIF-1α knockout resulted in lower NOX4 and higher NOX4C expression via alternative splicing and decreased the cell viability and increased the therapy sensibility in ovarian cancer cells.

### 3.3. IGF-1 Treatment Increased the Expression Levels of HIF-1α and NOX4 in A2780 and OVCAR3 Cells

**Figure 3:**
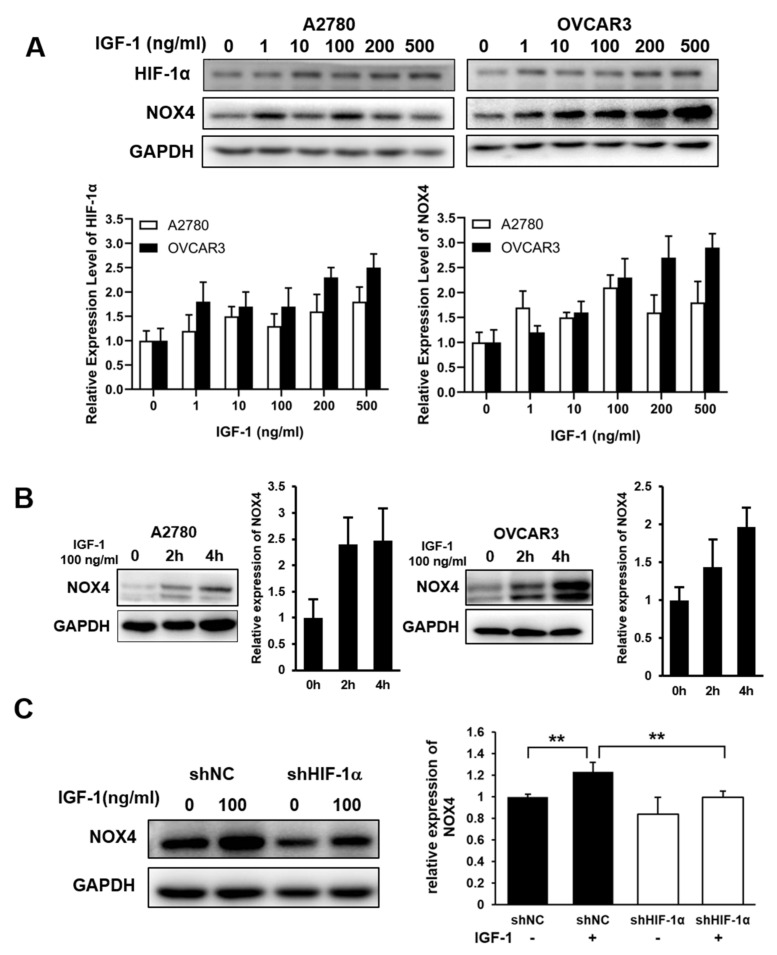
IGF-1 treatment increased the expression levels of HIF-1α and NOX4 in A2780 and OVCAR3 cells. (**A**) Cells were serum starved for 16 h, and treated with the indicated concentrations of IGF-1 for 4 h. The expression levels of HIF-1α and NOX4 were detected via Western blotting. (**B**) The serum-starved cells were treated with 100 ng/mL IGF-1 for 0 to 4 h, as indicated. The expression levels of NOX4 were analyzed by Western blotting. (**C**) The HIF-1α knockdown cells were serum-starved and treated with 100 ng/mL IGF-1 for 4 h, the expression levels of NOX4 were analyzed by Western blotting. ** indicates significant difference at *p* < 0.01.

IGF-1 plays a key role in the development, maintenance and chemotherapeutic response of ovarian cancer [12], and serum concentrations of free IGF-1 were significantly increased in ovarian cancer patients [13]. Therefore, we tested whether IGF-1 could induce the expression levels of HIF-1α and NOX4. A2780 and OVCAR3 cells were incubated with various concentrations of IGF-1 for 4 h; HIF-1α and NOX4 expression levels were analyzed by Western blot. At the range of physiologically relevant concentrations, IGF-1 markedly induced HIF-1α and NOX4 expression levels in human ovarian cancer A2780 and OVCAR3 cells (Figure 3A), thus HIF-1α might be one of the mechanisms regulating the expression level of NOX4. A2780 and OVCAR3 cells were incubated with IGF-I for 0, 2 or 4 h; NOX4 protein levels were induced by IGF-1 treatment in a time-dependent manner (Figure 3B). The cells were transfected with shHIF-1α and treated with 100 ng/mL IGF-1 for 4 h; NOX4 expression levels were then detected by Western blotting. IGF-1 treatment increased NOX4 expression by 23% in control shNC-transfected cells, while NOX4 expression levels were decreased by 24% in HIF-1α knockdown cells (Figure 3C). These results suggested that knockdown of HIF-1α significantly decreased NOX4 expression.

### 3.4. NOX4 Knockdown Increased the Sensitivity to Trastuzumab Treatment via Downregulating HER3 in Ovarian Cancer Cells

**Figure 4:**
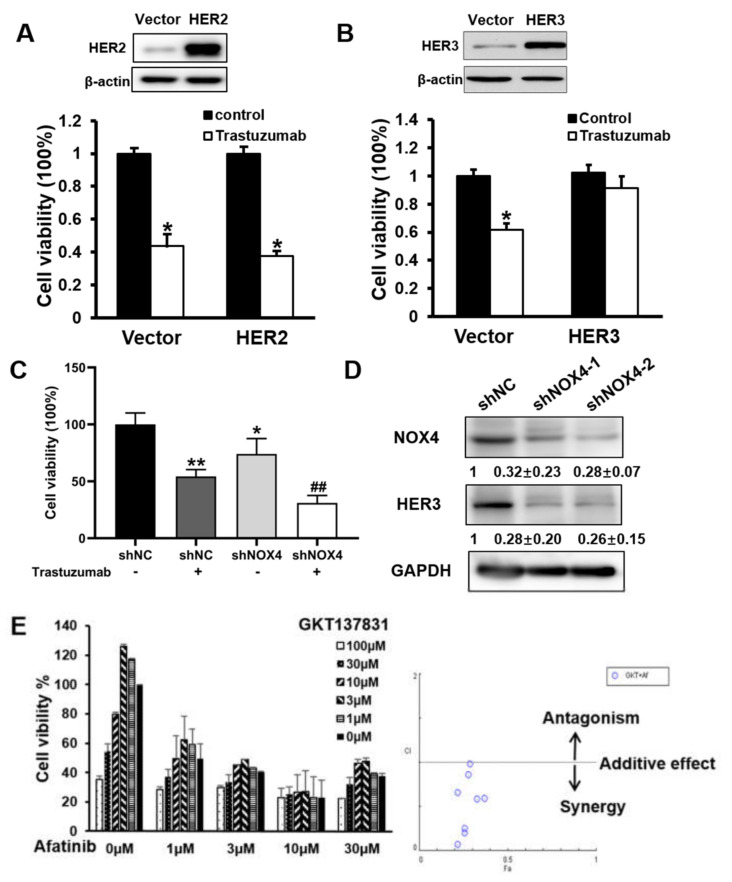
NOX4 knockdown increased the sensitivity to trastuzumab treatment via downregulating HER3 in ovarian cancer cells. (**A**,**B**) Overexpression HER2 and HER3 in A2780 cells; cells were treated with Trastuzumab and cell viability was detected via CCK8. (**C**) Knockdown of NOX4 in the cells via shNOX4; cells were treated with trastuzumab (100 mg/mL) for 4 days, and cell viabilities were detected by CCK8. * indicates significant difference at *p* < 0.05 compared with trastuzumab alone or shNOX4 group, ** indicates significant difference at *p* < 0.01 compared with trastuzumab alone or shNOX4 group, # indicates significant difference at *p* < 0.05, ## iindicates significant difference at *p* < 0.01. (**D**) The expression levels of HER3 in NOX4 knockdown cells were de-tected via Western blotting. (**E**) Combination treatment of NOX4 inhibitor (GKT137831) and afatinib; cell viability was detected after 72 h and CI values were calculated by software CompuSyn.

HER2 and HER3 expression was reported to be involved in drug resistance of ovarian cancer cells [14,15,16,17]. As shown in Supplementary Figure S1, the HER2 and HER3 expression levels were higher and highly correlated with the overall survival of ovarian cancer patients. To further evaluate whether forced HER2 or HER3 overexpression was sufficient to reverse drug resistance, A2780 cells were infected with lentivirus containing either HER2 cDNA without 3′-UTR (Figure 4A) or HER3 cDNA without 3′-UTR (Figure 4B), stable cell lines were obtained by puromycin selection. Cells were then treated with trastuzumab treatment and the effect on cell viability was determined. As shown in Figure 4A,B, trastuzumab treatment significantly reduced cell viability to 42% of the control in wild type cells. In HER2 over-expressing cells, trastuzumab treatment reduced cell viability to 36% of the control (Figure 4A), suggesting a small decrease in sensitivity to trastuzumab treatment. In contrast, in HER3 over-expressing cells, trastuzumab treatment did not significantly reduce cell viability compared with the control (Figure 4B), suggesting HER3 is an important molecule in drug resistance. In order to investigate whether NOX4 is involved in the targeted therapy resistance of ovarian cancer, we tested whether NOX4 knockdown affected the sensitivity of ovarian cancer to trastuzumab (HER2 inhibitor) treatment. A2780 stable cell lines with NOX4 knockdown were obtained through the shNOX4 lentivirus infection and puromycin selection. Cells were treated with trastuzumab, and cell viability was examined. As shown in Figure 4C, NOX4 knockdown significantly increased cell sensitivity to trastuzumab-induced cytotoxicity as compared with control shNC-transfected cells. To investigate whether NOX4-induced therapeutic resistance via HER3, we found that NOX4 knockdown significantly reduced HER3 expression levels (Figure 4D). Furthermore, NOX4 inhibitor (GKT137831) and another HER2 inhibitor, afatinib, produced a synergistic effect, as compared with GKT137831 or afatinib alone in A2780 cells (Figure 4E). These results suggested that the decreased expression of HER3, but not HER2, was the main reason for increased sensitivity of targeted therapy after NOX4 knockdown.

### 3.5. Knockdown of NOX4 and HIF-1α Increased Sensitivity to Radiation Therapy in Ovarian Cancer Cells

**Figure 5:**
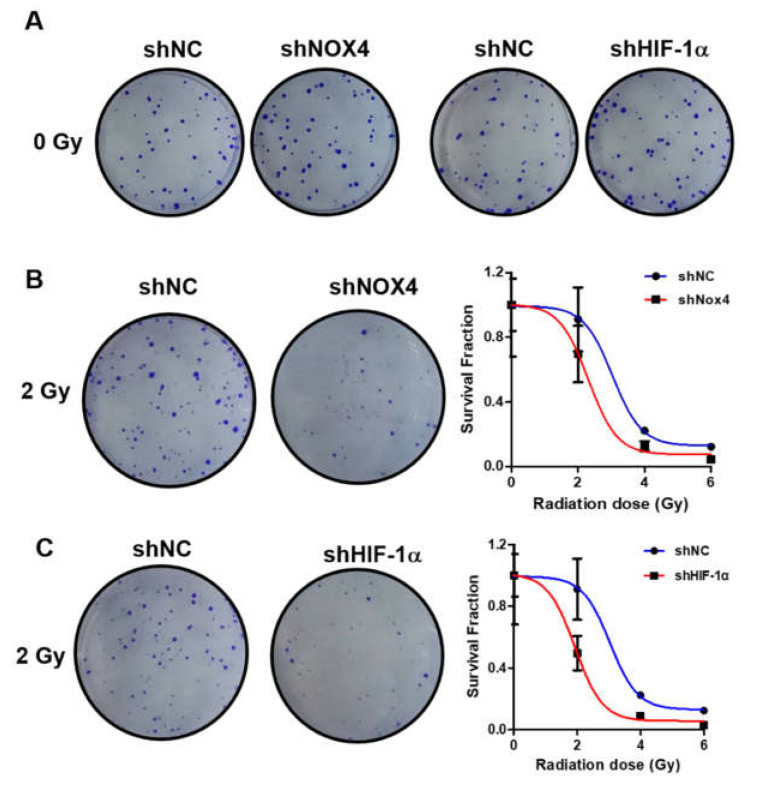
Knockdown of NOX4 and HIF-1α increased cell sensitivity to radiation treatment of cancer cells. (**A**–**C**) Knockdown of NOX4 and HIF-1α in OVCAR3 cells was performed using shNOX4 and shHIF-1α, radiation treatment was performed at 48 h after transfection. The number of cell clones were counted, and levels of survival cells were analyzed.

In order to explore the role of NOX4 and HIF-1α in the radiation therapy of ovarian cancer, OVCAR3 cells were transfected with shNOX4 or control shNC vector; at 48 h after transfection, cells were irradiated with 6MV X-ray linear accelerator at doses of 0 Gy, 2 Gy, 4 Gy and 6 Gy. Then, cells were plated in a 6-well plate and incubated for 10–14 days, the numbers of cell clones were counted, and survival cells were analyzed. The knockdown of NOX4 and HIF-1α had no significantly effect to the number of cell clones at 0 Gy (Figure 5A). As shown in Figure 5B, the number of cell clones significantly decreased in the NOX4 knockdown group, as compared with the control, indicating that NOX4 knockdown decreased cell survival and increased radiation sensitivity. Similarly, knockdown of HIF-1α by transfection of shHIF-1α also greatly increased radiation sensitivity as compared with the control (Figure 5C).

### 3.6. Knockdown of NOX4 Decreased the Transcriptional Activity and Expression Levels of NF-κB p65, and Increased Sensitivity to Afatinib Treatment in A2780 and OVCAR3 Cells

**Figure 6:**
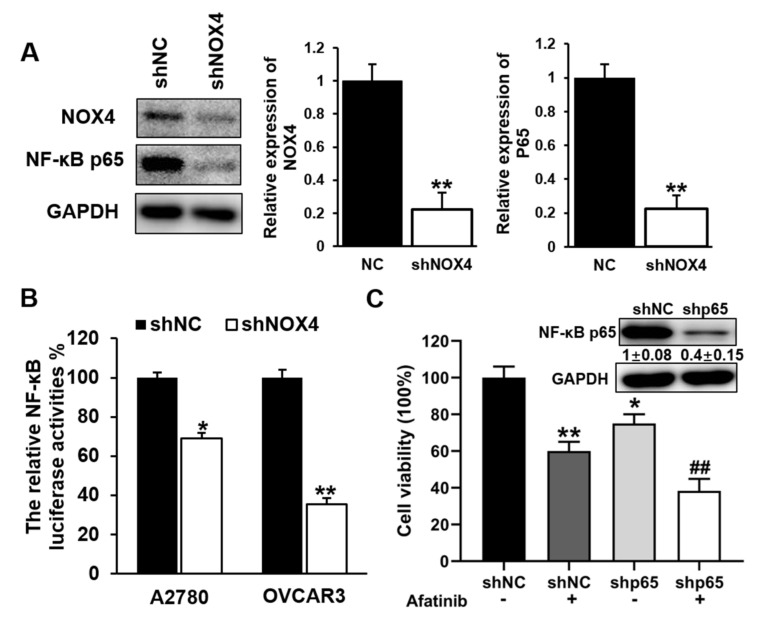
Knockdown of NOX4 in the cells decreased the transcriptional activity and expression level of NF-κB p65, and increased sensitivity to afatinib treatment in A2780 and OVCAR3 cells. (**A**) The expression levels of NF-κB p65 were detected by Western blotting in A2780 cells with knockdown of NOX4. (**B**) NF-κB p65 promoter luciferase reporter activities in NOX4 knockdown cells. (**C**) Cells were transfected with shNC or shp65 and treated with or without afatinib (3 μM/mL), the levels of survival cells were determined 4 days later. ** Indicates significant difference compared with shNC group at *p* < 0.01, and ## indicates significant difference at *p* < 0.01 compared with afatinib alone or shNOX4 group. ** Indicates significant difference at *p* < 0.01, * indicates significant difference at *p* < 0.05.

NF-κB p65 was reported to promote cell proliferation, invasion and migration in ovarian cancer [18,19]. To investigate whether NOX4 regulates expression of NF-κB p65, cells were transfected with either lentivirus carrying shNOX4 or control shNC, total proteins were extracted for Western blotting to analyze NF-κB p65 protein levels. As shown in Figure 6A, NOX4 knockdown significantly decreased NF-κB p65 expression in A2780 cells. We also measured NF-κB p65 transcriptional activity using its promoter luciferase reporter system. Compared with control, knockdown of NOX4 significantly decreased the relative luciferase activities of NF-κB p65 in both A2780 and OVCAR3 cells (Figure 6B). To explore whether NF-κB p65 regulated chemotherapeutic resistance, A2780 cells were transfected with lentivirus carrying NF-κB p65, and stable cell lines were obtained by puromycin selection. Afatinib was used to treat the cells and the survival cells were analyzed. Results showed that afatinib treatment decreased cell viability, and knockdown of NF-κB p65 expression further decreased cell viability with afatinib treatment (Figure 6D). These results suggested that NOX4 regulated the transcriptional activity and expression level of NF-κB p65, and further regulated sensitivity to afatinib treatment in A2780 and OVCAR3 cells.

## 4. Discussion

**Figure 7:**
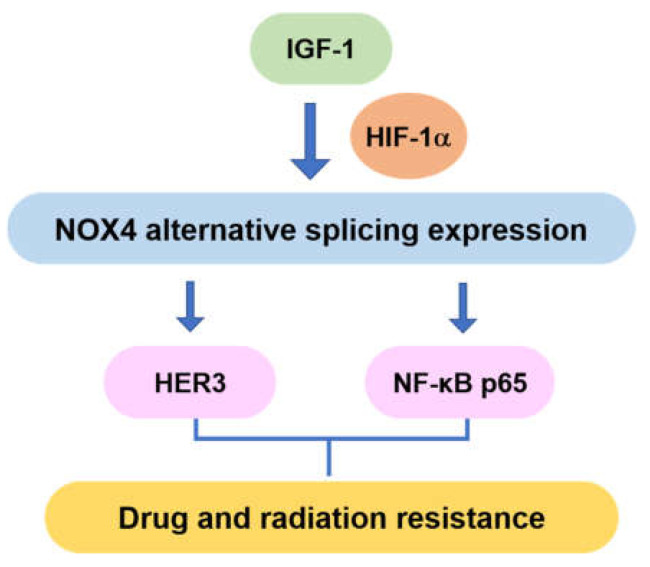
Overall model of NOX4 signaling in ovarian cancer.

Ovarian cancer is the most lethal malignancy among women, mainly due to its advanced stages when diagnosed, recurrence and resistance to current chemotherapeutic agents. It has been well documented that HIF-1 and vascular endothelial growth factor (VEGF) played important roles in the progression of ovarian cancer, angiogenesis and tumor growth [5]. We reported that ovarian cancer cells had higher expression levels of NOX4 [5]. In this study, as showed in the overall model of Figure 7, we showed that NOX4 expression levels were increased in human ovarian cancer tissues, compared with the adjacent tissues, and higher expression of NOX4 was associated with poor survival and progression of ovarian cancer. Interestingly, this study showed that HIF-1α directly regulated NOX4 expression, which is consistent with the previous report that NOX4 was induced by hypoxia [20,21]. NOX4 was reported to produce different isoforms by cutting off exons containing NAD(P)H/FADH binding regions. NOX4C is a variant splicing of exons from 9 to 11, which does not contain any NAD(P)H or FADH binding sites in proteins by decreasing ROS levels [10]. We found that HIF-1α knockdown induced higher expression levels of NOX4C.

Drug resistance is a complex phenomenon and the number of genes and signaling pathways may be involved in this process. Thus, better understanding of molecular mechanisms is crucial for management of ovarian cancer treatment [22]. HER2 monoclonal antibody trastuzumab has achieved exciting results in the treatment of breast cancer; however, Phase II clinical trials confirmed that trastuzumab is not effective in the treatment of ovarian cancer [8], which may be due to this novel HIF-1/NOX4 pathway.

In this study, we demonstrated that overexpression of HER3, but not HER2, might be a main reason why trastuzumab was not effective in the treatment of ovarian cancer. Previously, we revealed that ROS induced HER2 and HER3 expression in ovarian cancer cells [23]; here, we found that NOX4 knockdown decreased the expression level of HER3, suggesting that NOX4 regulated drug resistance through HER3. We showed that, while overexpression of HER2 slightly decreased the sensitivity to trastuzumab treatment, overexpression of HER3 greatly increased the resistance to trastuzumab treatment.

In this study, we also found that combination therapy of NOX4 inhibitor (GKT137831) and afatinib decreased cell viability and had a synergy effect in ovarian cancer cells, which might be a strategy for ovarian cancer treatment. Afatinib, also known as BIBW2992, was shown to have significant benefits in progression-free survival when compared with standard doublet chemotherapy in NSCLC patients with EGFR mutations in two randomized Phase III trials [24,25]. Afatinib is also a therapeutic option as a HER2-targeted therapy for NSCLC harboring HER2 amplification or mutations [26]. It has been approved for the treatment of EGFR-mutant NSCLCs in several countries. The combination of paclitaxel and afatinib was found to induce significant tumor regressions and tumor necrosis of the A2780 xenografts [27].

In addition to drug resistance, we found that NOX4 knockdown increased radiation sensitivity, which is consistent with the study that NOX4 blockage enhances the efficiency of radiotherapy in glioblastoma multiforme xenografts [28]. Moreover, HIF-1α knockdown also greatly increased radiation sensitivity, indicating that HIF-1α was an effector of NOX4 for mediating radiation resistance in ovarian cancer cells. Thus, this finding may be a potential new mechanism of therapeutic resistance with clinical implications in other cancers.

Taken together, these results indicated that NOX4 may be a novel prognostic marker and therapeutic target for ovarian cancer. The downstream genes of NOX4, such as HER3 or/and NF-κB, may play important roles in the regulation of therapeutic sensitivity, which may provide an experimental basis for further investigation into improving ovarian cancer treatment and prognosis in the future.

## Data Availability

TCGA (The Cancer Genome Atlas) database at https://xenabrowser.net/datapages/, accessed on 30 June 2021.

## Supplementary Materials

The following are available online at https://www.mdpi.com/article/10.3390/cells10071647/s1, Figure S1: The HIF-1α, HER2 and HER3 expression levels.
