# Supplementary figures and images for: NOX4 Signaling Mediates Cancer Development and Therapeutic Resistance through HER3 in Ovarian Cancer Cells
Source: Cells. 2021 Jun 30;10(7):1647. doi: 10.3390/cells10071647 (PMC8304464; doi:10.3390/cells10071647)

## Slide 1
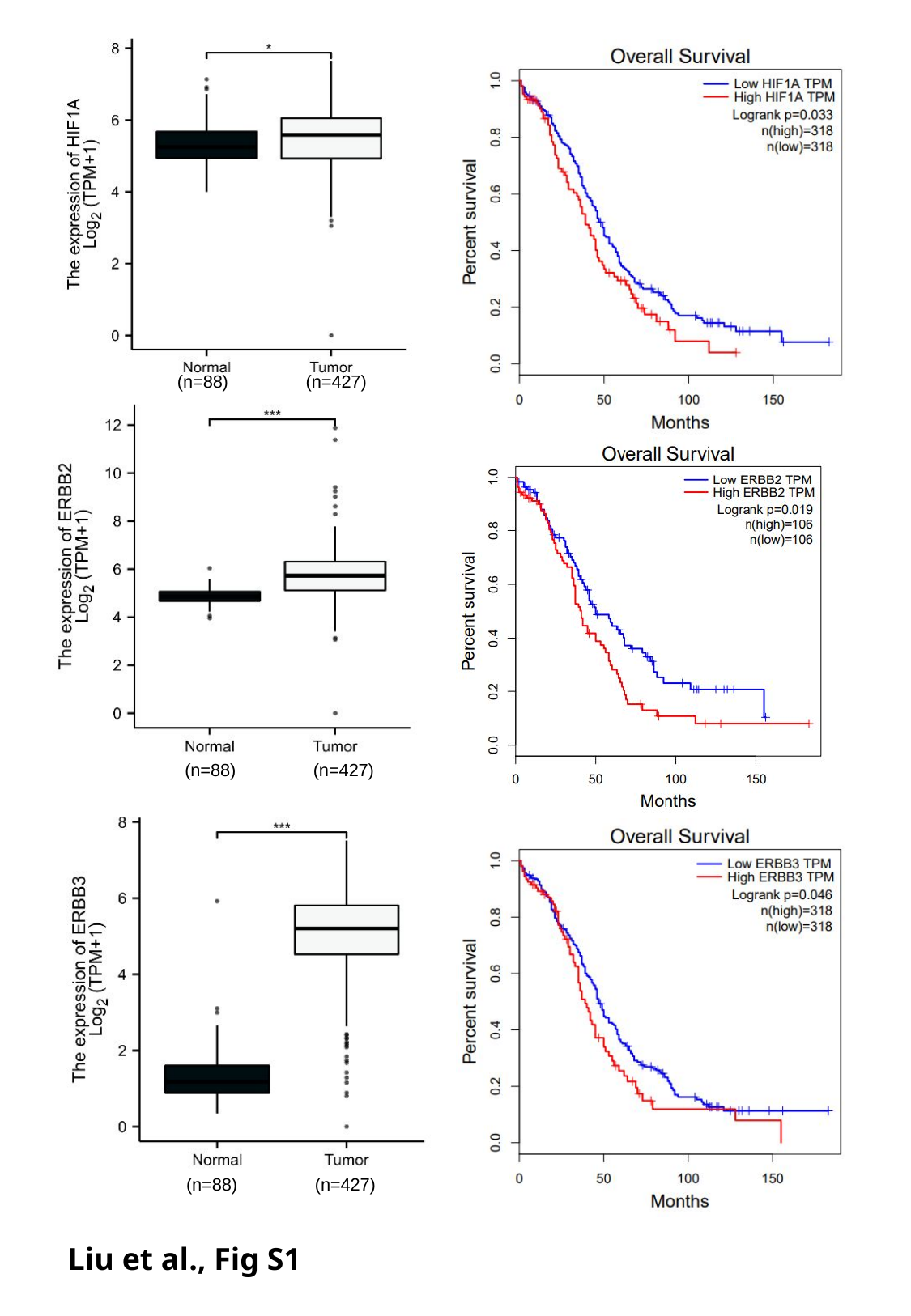

(n=88)
(n=427)
(n=88)
(n=427)
(n=88)
(n=427)
Liu et al., Fig S1

Supplement: Supplementary file 1 [file cells-10-01647-s001.zip › Supplementary Files/Supplementary Data.pptx]
